# Perirectal Abscess Masquerading as Cauda Equina Syndrome in an Otherwise Healthy 12-Year-Old Child

**DOI:** 10.1155/2014/817124

**Published:** 2014-02-13

**Authors:** Dylan Dean

**Affiliations:** Center for Policy and Emergency Medicine Research, Oregon Health and Science University, 3181 SW Sam Jackson Boulevard, Portland, OR 97239, USA

## Abstract

A 12-year-old boy was brought to an urgent care center for fever, back pain, and abnormal gait. In addition to back pain, the patient was found to be persistently febrile but also had decreased perianal sensation and bowel incontinence. He was therefore referred to the emergency department where his back pain improved without medication but he was still febrile with bowel incontinence and persistently decreased perianal sensation. An MRI was ordered to evaluate possible cauda equina syndrome and revealed a perirectal abscess. The child ultimately underwent an exam under anesthesia with pediatric surgery and had a drain placed. This case highlights a unique presentation of perirectal abscess masquerading as cauda equina syndrome. A discussion of important considerations in emergency room diagnosis and management is presented.

## 1. Introduction

Cauda equina syndrome due to epidural abscess should be high in the differential diagnosis of patients presenting with fever, back pain, decreased perianal sensation, and bowel incontinence but other disease processes can masquerade as this spinal cord pathology. Presented is to our knowledge the only case of a perirectal abscess in a preadolescent child presenting with exam findings suggestive of cauda equina syndrome.

## 2. Case Presentation

A 12-year-old otherwise healthy boy was referred from an urgent care center to our emergency department for fever and back pain. Per telephone report from the referring facility, the child had had a few days of leg pain and lumbar spine pain and was febrile to 102°F. A rectal exam was performed during which the child was reportedly incontinent of “bloody-brown stool.”

The patient arrived by ambulance to our emergency department in no acute distress. He complained of bilateral lower leg pain over the past week that had started laterally in his left hip, was achy, radiated to his lower legs but not to his feet, and was worse with movement and better somewhat with rest. His mom reported that he had abnormal gait. By the day of evaluation, he had developed focal lower back pain that was achy, sometimes sharp, worse with movement and sitting but better when supine. He had also been febrile to 101°F at home prompting his mother to bring him to urgent care. He had had no urinary retention associated with his back pain although he did have the above reported bowel incontinence. The patient stated that he could not feel the rectal exam at the urgent care center nor sense that he had been incontinent of stool until he was told that he had been so. Of note, he emphasized that his leg pain had almost completely resolved after digital rectal examination but that his back pain, although improved, was still present. He had had some loose stools in the past two days.

After being triaged to an acute care room, the patient's initial vital signs were Temp 37.1°C, HR 95, BP 103/57, RR 12, O2 sats 96% on room air. He was generally well-appearing and thin. His head/eyes/ears/nose/throat exams were normal. He had no neck pain, stiffness, or meningismus, and he had normal cardiovascular and pulmonary exams. His abdomen was soft and nontender. He had no costal-vertebral angle tenderness to palpation but did complain of midline low back pain to palpation. His arms and legs were without acute abnormality. He had no abnormal skin findings. His neurologic examination was notable for increased tone in his quadriceps bilaterally although he was able to relax with significant coaching. Otherwise, he had a normal cranial nerve exam, normal gait, normal toe and heel walking, 5/5 motor strength proximally and distally throughout, normal sensation to light touch throughout, normal speech, no dysmetria.

When the patient rolled to his side for a digital rectal exam, he was found to have copious amounts of stool-like, oozing, thick, brownish-red, malodorous fluid exuding from his anus. After cleaning him, his external anal exam was normal-appearing. A digital exam was performed and was negative for mass although he was unable to sense the exam or the fluid exuding from his anus. He was able to bear down.

An IV was placed and blood work was obtained. His laboratory studies were notable for mild leukocytosis to 13,000 without left-shift and complete metabolic panel without acute abnormality. “Stool” was sampled and found to be guaiac positive. Urine studies were normal and postvoid residual volume was less than 100 cc.

**Figure 1:**
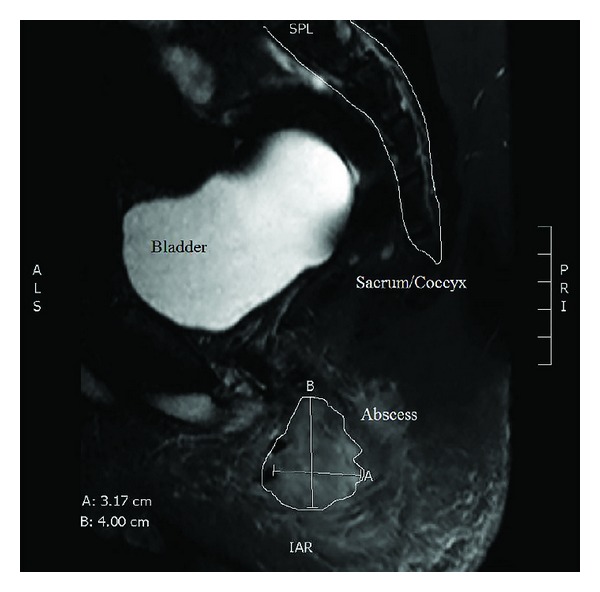
T2 MRI, sagittal view sacrum/coccyx. Magnetic resonance imaging revealing an approximately 3.0 × 4.0 cm phlegmon consistent with perirectal abscess.

An MRI of the lumbar spine was obtained and was negative for spinal abnormality but notable for perirectal phlegmon (Figure 1). Together with our physical exam findings and the child's history of fever, this was felt to be diagnostic for a perirectal abscess. Interestingly, the pain from the fluid collection had apparently been relieved by digital rectal which had likely caused spontaneous drainage and decompression.

Ultimately, the child was taken to the operating room with pediatric surgery for exam under anesthesia due to recurrence of pain. The abscess, draining rectally, was confirmed by exam. The residual fluid collection was drained and a 10-French Malecot catheter was placed, flushed to ensure functionality and sutured in place. On discharge, two days after initial evaluation, the child was eating, drinking, and ambulating well. He is to follow up with pediatric surgery subsequently in the outpatient setting.

## 3. Discussion

Described in this case report is a 12-year-old child who had fever, back pain, and leg pain and reported bowel incontinence and decreased perianal sensation, but whose underlying etiology was a perirectal abscess as opposed to epidural abscess causing cauda equina syndrome.

Perirectal abscesses, although considered common in younger children, are relatively infrequent in 12-year olds and are considered to be indicators of underlying inflammatory bowel disease or other serious pathologies [1, 2]. They are even rare as presenting pathology in the diagnosis of Crohn's disease, occurring in only 2% to 13% of patients, which may become relevant for our patient who had not had prior symptoms of inflammatory bowel disease [3, 4]. Children with perirectal abscesses most frequently present with fever, rectal pain or mass, pain with sitting or defecation, and even abnormal gait [1] but not with back pain and decreased perianal sensation, as in the case of our patient.

Our case suggests that MR imaging could be the appropriate initial imaging modality to distinguish between cauda equina and perirectal abscess. This is important in that appropriate imaging modality for diagnosing perirectal abscesses alone in children is somewhat controversial. MRI, CT, and endosonography have been shown to be similarly accurate tests [5] although a study of 22 adults showed that anal endosonography with a linear probe was more accurate than even MRI in detecting anorectal abscesses in Crohn's disease patients [6]. CT imaging is intuitively less favorable in children due to increased risks of adverse effects due to radiation. An interesting future study based on this case would be the evaluation of the utility of emergency physician-performed bedside ultrasound in diagnosis of perirectal abscess, particularly in children.

Our child had findings to suggest that his abscess had rectal communication which we felt precluded emergency department intervention. However, management of perirectal abscesses is somewhat controversial. In adults, some data support early drainage and antibiotics in the emergency department [7]. Although incision and drainage are indeed considered standard management [8], children who undergo these procedures have been shown to have fistula-in-ano or recurrence in up to 50% of cases [9]. One study involving 91 children over 13 years showed that drainage and laying open of fistulas associated with abscesses resulted in decreased abscess recurrence [10] although placement of draining setons has been shown to be more appropriate than fistulotomy [11]. Nonoperative management has been demonstrated to be effective but with prolonged courses and less frequent resolve [4, 12]. Importantly, perirectal abscesses have been reported to progress to Fournier's Gangrene if not monitored appropriately [13]. Further study is needed to evaluate criteria for appropriate emergency department intervention, particularly in children.

Our case in emergency department management is a reminder to keep the differential diagnosis broad, as perirectal abscesses—particularly in children—can masquerade as other pathologies. Our patient's symptoms were initially concerning for cauda equina syndrome due to epidural abscess; however, there was no history of urinary retention which was a key component of the history. What was felt to “bowel incontinence” was actually drainage of copious, foul-smelling, stool-like, purulent fluid. It is still unclear why our patient was unable to sense either rectal exam or drainage of fluid. Beyond that of cauda equina syndrome, another masquerading presentation to consider is sexual abuse as demonstrated in one study with 267 children with Crohn's and no history of abuse in whom up to 41% had perianal erythema in both males and females and perianal tags in up to 11% of females [14].

Beyond emphasizing the importance of a broad differential diagnosis, our case highlights the importance of the rectal exam in fever and back pain in children. Although back pain has become more prevalent in children, it is still considered a red flag for pathologic process [15]. As in our case, the rectal exam can even be both diagnostic and therapeutic if causing drainage of the abscess. If there is any confusion between purulent fluid and stool, a gram stain would most likely be diagnostic. Further, MRI studies can evaluate both spinal cord pathology and perirectal abscesses while avoiding possibly unnecessary radiation of a child although bedside ultrasound could be an important future tool for emergency physicians. Although incision and drainage, with or without drain placement or antibiotics, remains the recommended treatment, further study is needed to elucidate optimal management in the emergency department.
